# The effect of asymmetric movement support on muscle activity during Lokomat guided gait in able-bodied individuals

**DOI:** 10.1371/journal.pone.0198473

**Published:** 2018-06-04

**Authors:** Sylvana Weiland, Ineke H. Smit, Heleen Reinders-Messelink, Lucas H. V. van der Woude, Klaske van Kammen, Rob den Otter

**Affiliations:** 1 University of Groningen, University Medical Center Groningen, Center for Human Movement Sciences, Groningen, The Netherlands; 2 Rehabilitation Center 'Revalidatie Friesland', Beetsterzwaag, The Netherlands; 3 University of Groningen, University Medical Center Groningen, Center for Rehabilitation, Groningen, The Netherlands; University of Colorado Boulder, UNITED STATES

## Abstract

**Background:**

To accommodate training for unilaterally affected patients (e.g. stroke), the Lokomat (a popular robotic exoskeleton-based gait trainer) provides the possibility to set the amount of movement guidance for each leg independently. Given the interlimb couplings, such asymmetrical settings may result in complex effects, in which ipsilateral activity co-depends on the amount of guidance offered to the contralateral leg. To test this idea, the effect of asymmetrical guidance on muscle activity was explored.

**Methods:**

15 healthy participants walked in the Lokomat at two speeds (1 and 2 km/h) and guidance levels (30% and 100%), during symmetrical (both legs receiving 30% or 100% guidance) and asymmetrical conditions (one leg receiving 30% and the other 100% guidance) resulting in eight unique conditions. Activity of the right leg was recorded from Erector Spinae, Gluteus Medius, Biceps Femoris, Semitendinosus, Vastus Medialis, Rectus Femoris, Medial Gastrocnemius and Tibialis Anterior. Statistical Parametric Mapping was used to assess whether ipsilateral muscle activity depended on guidance settings for the contralateral leg.

**Results:**

Muscle output amplitude not only depended on ipsilateral guidance settings, but also on the amount of guidance provided to the contralateral leg. More specifically, when the contralateral leg received less guidance, ipsilateral activity of Gluteus Medius and Medial Gastrocnemius increased during stance. Conversely, when the contralateral leg received more guidance, ipsilateral muscle activity for these muscles decreased. These effects were specifically observed at 1 km/h, but not at 2 km/h.

**Conclusions:**

This is the first study of asymmetrical guidance on muscle activity in the Lokomat, which shows that ipsilateral activity co-depends on the amount of contralateral guidance. In therapy, these properties may be exploited e.g. to promote active contributions by the more affected leg. Therefore, the present results urge further research on the use of asymmetrical guidance in patient groups targeted by Lokomat training.

## Introduction

For persons with motor impairments due to neurological disease or trauma, the ability to walk is an important determinant of independent functioning [1]. Clinical evidence shows that the major determinants of functional outcome are task specificity and training intensity: in order to re-learn to walk, patients have to be involved in walking activity and produce a large number of stepping movements [2,3]. In the past decade, robotic gait trainers have been developed that eliminate the need for manual assistance by therapist, as actuated exoskeletons allow automated support during more stepping movements. As studies on the clinical effectiveness of robot-assisted gait trainers (RAGT) thus far have yielded positive (see e.g. [4]) as well as negative results (see e.g. [5]), there is a need to more fully understand how training can be shaped through parameter settings (e.g. the amount of guidance), and to explore the specific possibilities offered by RAGT.

RAGTs have the unique ability to provide automated movement support (so-called ‘guidance’), and guide the legs through a predefined gait pattern [6]. A popular treadmill-based robotic device for gait training is the Lokomat [6], which is able to offer guidance based on two possible strategies: path control and impedance control [7,8]. In this study, the impedance control strategy was used, which allows adjustable control over the amount of supportive force that ‘guides’ the legs through the gait cycle [8]. A significant proportion of the target population for RAGT (e.g. stroke, cerebral palsy) suffers from asymmetrical locomotor impairments, and would therefore require more assistance for one leg than for the other. To accommodate these training needs, guidance levels in the Lokomat can be set separately for each limb, making it possible to tune the required contributions specifically to the capacity of each leg. Although the neuromuscular control of Lokomat guided gait [9–11] and the effects of symmetrical guidance [12,13] have been described in good detail, it is still unclear how the muscle activity that drives Lokomat guided gait is affected when guidance is provided asymmetrically.

Arguably, under asymmetrical conditions, the involvement of muscles may not only depend on the guidance offered to the ipsilateral leg, but also on the amount of guidance provided to the contralateral leg. Stable bipedal progression is organized bilaterally, and depends on the coupled control of the two legs. Studies in which unequal contributions of the legs are required show that the phasing and amplitude of ipsilateral muscle activity depend on the activity of the contralateral leg [14–20]. These couplings likely involve networks that integrate ascending (e.g. hip afferents and load receptors) and descending (spinal and supra-spinal) commands [21]. The bilateral involvement of these networks can be demonstrated quite dramatically in tasks where movements of one of the legs are blocked. Under these conditions, structurally phased activity in the passive leg can still be induced through cyclical, task specific movements of the contralateral leg [17,18,21–23]. Observations like these suggest that levels of gait specific neuromuscular activity in the ipsilateral leg may be tuned to the contributions made by the contralateral leg.

Previous research on Lokomat guided gait has shown that the contribution of muscles is inversely related to the amount of symmetrical guidance that is provided, so that higher levels of guidance generally induce lower levels of muscle activity [12,13]. It can be argued that, when guidance is provided asymmetrically, varying the level of contralateral guidance may alter the activity of ipsilateral muscles. For therapists, this may open up new opportunities to stimulate more active participation of patients during training. To test these ideas in the present study, able-bodied subjects walked in the Lokomat under symmetrical as well as asymmetrical guidance conditions. The aim was to assess if and to what extent levels of ipsilateral muscle activity depend on the level of guidance provided to the contralateral leg. As previous work has shown that the effects of guidance depend on gait speed [12], a secondary aim was to assess if the effects of asymmetrical guidance depend on treadmill speed. To this end, muscle activity was assessed in healthy participants during symmetrical and asymmetrical guidance conditions at both 1 and 2 km/h.

## Methods

### Participants

Fifteen healthy adults (5 male, 22.2 ± 2.1 year, 1.73 ± 0.07 meter and 65.6 ± 9.52 kg) participated in this study. None of the subjects had a condition that is known to affect the gait pattern or muscle activity. All experimental procedures were approved by the Ethical Committee of the Center for Human Movement Sciences (University Medical Center Groningen, the Netherlands) and performed according to the declaration of Helsinki for medical research involving human subjects [24]. All subjects provided written informed consent prior to the procedure.

### Materials

Participants walked in the LokomatPro V6.0 (Hocoma AG, Volketswil, Switzerland) at the rehabilitation center ‘Revalidatie Friesland’ in Beetsterzwaag, the Netherlands. The Lokomat is a stationary gait orthosis that moves the legs through the gait cycle by means of actuated knee and hip joints that are integrated into the exoskeleton [8]. The Lokomat offers the opportunity to set the level of guidance, body weight support (BWS) and treadmill speed. In the present study, guidance was provided by means of an impedance controller, which applies a controllable torque to each joint to keep the legs on the reference trajectory [7,8]. When guidance is reduced, a smaller torque that pushes the legs towards the pre-defined trajectory is applied compared to maximal guidance [8].

Surface electromyography (EMG) was used to record muscle activation patterns from eight muscles of the right (ipsilateral) leg: Erector Spinae (ES), Gluteus Medius (GM), Biceps Femoris (BF), Semitendinosus (ST), Vastus Medialis (VM), Rectus Femoris (RF), Medial Gastrocnemius (MG) and Tibialis Anterior (TA). Ag/AgCl electrodes (Kendall/Tyco ARBO; Warren, MI, USA) with a 10 mm diameter and a minimum electrode distance of 25 mm, were used and placed following the SENIAM recommendations [25]. To ensure good conduction, body hair and dead skin cells were removed by shaving and abrading the electrode sites, whereupon these sites were cleaned with alcohol. EMG signals were pre-amplified and A/D converted (22 bits) using a 32-channel Porti7 portable recording system (Twente Medical Systems, Enschede, The Netherlands). The recording system has a common mode rejection of 0.90 dB, a 2 mVpp noise level and an input impedance of 0.10 GV. To detect gait cycle events, custom made insoles (Pedag international VIVA, Berlin, Germany) were used containing one pressure sensor under the heel and three pressure sensors under the forefoot (FSR402, diameter 18 mm, loading 10–1000 g). The EMG and pressure sensor data were simultaneously sampled via the Porti7 system at 2048 Hz before storage on a computer for offline processing, so that no synchronization was required.

### Procedure

Participants walked sixteen trials in the Lokomat without BWS, and without foot straps, while guidance (30% and 100%) and treadmill speed (1 and 2 km/h) were varied. This resulted in symmetrical trials where both legs received 30% guidance (30 ipsilateral (IL) / 30 contralateral (CL)) or 100% guidance (100IL/100CL) and asymmetrical trials where contralateral guidance was higher (30IL/100CL) or lower (100IL/30CL) compared to ipsilateral guidance at both speed levels (see Table 1). To prevent order effects, a randomized order of the eight unique conditions (30IL/30CL, 100IL/100CL, 30IL/100CL and 100IL/30CL, at both 1 and 2 km/h) was presented first (block 1). Subsequently, the same conditions were presented a second time in a counter-balanced order (block 2) to control for learning and fatigue. To ensure that an approximately equal number of steps was measured, trials at 1 km/h lasted 120 seconds and trials at 2 km/h 60 seconds. The quality of the EMG and pressure sensor signals was constantly monitored during the experiment. Before starting each trial, an acclimatization period of two minutes was provided to the participants to get accustomed to the specific Lokomat settings.

**Table 1:** The conditions offered to the participants twice.

	30% guidance ipsilateral		100% guidance ipsilateral	
	Symmetrical condition	Asymmetrical condition	Symmetrical condition	Asymmetrical condition
1 km/h	*30IL/30CL*	*30IL/100CL*	*100IL/100CL*	*100IL/30CL*
2 km/h	*30IL/30CL*	*30IL/100CL*	*100IL/100CL*	*100IL/30CL*

Both legs received 30% guidance (30 ipsilateral (IL) / 30 contralateral (CL)) or 100% guidance (100IL/100CL) during symmetrical trials and a higher (30IL/100CL) or lower (100IL/30CL) level of contralateral guidance during asymmetrical trials.

### Data analysis

EMG and pressure sensor data were analysed offline using custom-made software routines in Matlab (Matlab 2015a; Mathworks, Natick, MA). The EMG data were filtered using a 10 Hz fourth order Butterworth high-pass filter to attenuate movement artefacts. Subsequently, EMG data were full wave rectified, low-pass filtered using a 20 Hz fourth order Butterworth filter, time normalized (from heelstrike to heelstrike based on insole pressure data) to 100 data points per gait cycle, and amplitude normalized with respect to the percentage of the maximal amplitude observed over all of the trials, for each muscle and each participant separately. For the analysis, muscle activity of the corresponding trials performed in block 1 and 2 were averaged.

### Statistical analysis

One approach to the statistical analysis of dynamic EMG is to compare the (summed or averaged) amplitudes within pre-specified time windows, e.g. the single stance phase, the swing phase and the first and second double support phase [12], or within specific time windows that are set arbitrarily based on the observed profiles [11]. However, previous research on muscle activity in the Lokomat has shown that activity may occur within windows that are a-typical for unrestrained overground or treadmill walking [11]. As this makes it difficult to define time windows a priori, we choose to assess the averaged and time-normalized EMG time series using Statistical Parametric Mapping (SPM).

SPM repeated measures ANOVA's (RM) were used to assess the effects of the factors Guidance (30 and 100%) and Symmetry (asymmetrical and symmetrical) for both speed levels, and all eight muscles, separately. SPM was originally developed to deal with the spatial correlation inherent in neuroimaging data [26], and has quite recently also been applied to biomechanical data (e.g. [27]). The output of an SPM RM analysis is a ‘Statistical Parametric Map’ that consists of F-values (SPM{F}) calculated separately for each data point *t* in the time normalized gait cycle (t = 1 …100). This implies that no time windows need to be defined a priori. Since there is a spatial correlation present in the smoothed EMG signal, the number of independent tests does not equal the number of data points in the time normalized gait cycle. To maintain the familywise error rate at the intended 5%, SPM estimates the trajectory smoothness using temporal gradients [28]. Based on these smoothness estimates and the number of data points, the actual number of independent tests can be determined. The required thresholds are then calculated based on an a priori alpha value (0.05 in this case) using Random Field Theory (RFT) expectations with regard to the field-wide maximum [29].

F-values that exceed the calculated threshold often occur in clusters of data points, representing significant segments of the time series in which less than 5% of smooth random curves would be expected to cross. The exact probability that a cluster of a specific size crosses the threshold can be calculated for each of these supra-threshold clusters, based on cluster size and RFT distribution(s) for SPM{F} topology. It is important to note here that SPM eliminates the need for a priori decisions with regard to regions of interest, and thus avoids potential bias due to e.g. data driven window setting. For a more in-depth discussion of SPM and its applications, we refer to more elaborate texts on this topic [26–28].

To be able to answer the research question, the effects of contralateral guidance on ipsilateral muscle activity (referred to as the asymmetrical guidance effects) were assessed by interpreting the interaction effects of Guidance by Symmetry. In addition, assessment of the main effects of Guidance (referred to as the general effects of guidance) allowed us to establish if and how possible effects of asymmetrical guidance are related to guidance induced inhibitions/facilitations of ipsilateral activity. Supra-threshold clusters for the main effect of Guidance indicate a difference in the amplitude normalized, ipsilateral muscle activity between 30 and 100% guidance conditions. Supra-threshold clusters for Guidance by Symmetry interaction indicate that the effects of guidance on the amplitude-normalized EMG values in the ipsilateral leg, depend on the level of guidance provided to the contralateral leg. In case of supra-threshold clusters, the direction of the effects was determined by comparing the condition means within the cluster. All SPM analyses were done using open-source spm1d code (v.M0.1, www.spm1d.org) in Matlab (R2016a, The Mathworks Inc, Natick, MA).

## Results

### General effects of guidance on muscle activity

Significant main effects of Guidance were found at 1 km/h, indicating that the level of ipsilateral muscle activity was negatively associated with the level of ipsilateral guidance that was provided. Muscle activity in the ipsilateral leg decreased when guidance increased from 30% to 100% in four supra-threshold clusters in ES (51.8–52.3%, p = 0.0496; 53.4–54.6%, p = 0.0477; 58.2–63.0%, p = 0.0256; 63.0–70.7%, p = 0.0084; critical threshold (CT) = 13.5 for all clusters) two supra-threshold clusters in BF (61.8–62.3%, p = 0.0495; 63.8–65.8%, p = 0.0407; CT = 14.0), two supra-threshold clusters in ST (13.8–14.1%, p = 0.0497; 17.7–18.4%, p = 0.0490; CT = 14.1), and one supra-threshold cluster in RF (79.6–85.6%, p = 0.0152; CT = 13.6). Similar main effects of guidance were observed at 2 km/h for ST as indicated by one supra-threshold cluster (96.0–96.0%, p = 0.0500; CT = 14.4). No other significant main effects of guidance were observed at 2 km/h.

### Effects of the Guidance by Symmetry interaction on muscle activity

**Fig 1:**
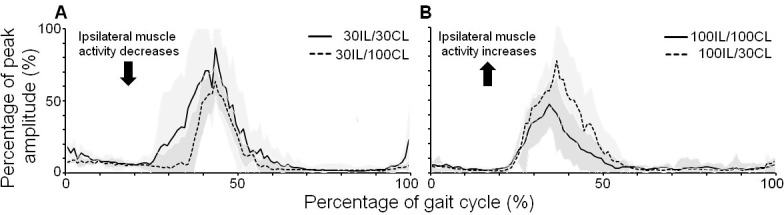
**EMG profiles for Medial Gastrocnemius (MG) when guidance offered to the contralateral leg (CL) increases (A) or decreases (B) while guidance of the ipsilateral leg (IL) was held constant at 30% (A) or 100% (B).** Time and amplitude normalized EMG profiles during walking in the Lokomat exoskeleton for a representative participant at 1 km/h. EMG amplitude is expressed as a percentage of peak amplitude recorded over all conditions. The standard deviation of the EMG profiles for each condition is presented by the dark shade.

Results showed that at 1 km/h ipsilateral muscle activity of GM and MG depended on the level of guidance provided to the contralateral leg. Fig 1 presents EMG profiles for MG of a single representative participant at 1 km/h to illustrate the effects. As presented in this figure, the amount of ipsilateral muscle activity depends on the amount of contralateral guidance. When ipsilateral guidance was set to 30%, the amplitude of muscle activity decreased when the contralateral leg was provided 100% guidance (30IL/100CL), compared to the symmetrical 30IL/30CL condition (see Fig 1B). An opposite effect was observed when ipsilateral guidance was set to 100%, as ipsilateral activity increased when contralateral guidance was lower (100IL/30CL) compared to the symmetrical 100IL/100CL condition (see Fig 1B).

**Fig 2:**
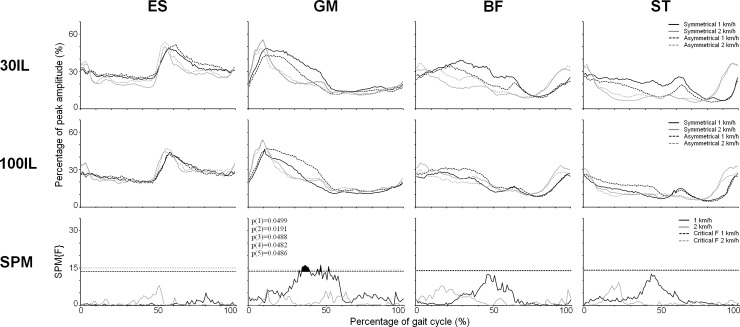
Average EMG profiles for Erector Spinae (ES), Gluteus Medius (GM), Biceps Femoris (BF) and Semitendinosus (ST) combined with the SPM results of the SPM analyses for the Guidance by Symmetry interaction. Averaged EMG profiles are presented for Erector Spinae (ES), Gluteus Medius (GM), Biceps Femoris (BF) and Semitendinosus (ST) combined with the results of the SPM analyses for the Guidance by Symmetry interaction. The EMG profiles are time and amplitude normalized during walking in the Lokomat exoskeleton with 30% guidance provided to the ipsilateral leg (first row) and 100% guidance provided to the ipsilateral leg (second row) at 1 km/h (black lines) and 2 km/h (grey lines) for both symmetrical (solid lines) and asymmetrical (dashed lines) conditions. EMG amplitude is expressed as a percentage of peak amplitude recorded over all conditions. The third row of figures display the SPM{F} values of the Guidance by Symmetry interaction at 1 km/h (black lines) and 2 km/h (grey lines). Threshold F values are indicated by dashed black lines (1 km/h) and dashed grey lines (2 km/h). The displayed p-values represent the chance that a supra-threshold cluster of the given cluster size would be observed in random samplings.

Group averaged EMG profiles are presented together with the SPM results of the interaction effect of Guidance by Symmetry in Fig 2 (ES, GM, BF and ST) and 3 (VM, RF, MG and TA). All supra-threshold clusters indicate segments of the EMG time series in which less than 5% of smooth random curves would be expected to cross the threshold (df = 1,14). As becomes clear from these figures, at 1 km/h one supra-threshold cluster (38.8–49.5% of the gait cycle, CT = 13,8; p = 0.0008) was identified for the Guidance by Symmetry interaction in MG. Comparison of the group-averaged values within this cluster showed that when contralateral guidance was higher (30IL/100CL), ipsilateral activity decreased compared to when both legs received low levels of guidance (30IL/30CL). In contrast (see Fig 2), when contralateral guidance was lower (100IL/30CL), ipsilateral activity increased relative to when both legs received maximal guidance (100IL/100CL). Similar effects of asymmetrical guidance were present in GM at 1 km/h as indicated by five supra-threshold clusters (31.9–32.2%, p = 0.0499; 33.5–39.0% p = 0.0191; 43.5–44.4%, p = 0.0488; 45.4–46.5%, p = 0.0482; 50.6–51.6% p = 0.0486, CT = 13.6 for all clusters).

**Fig 3:**
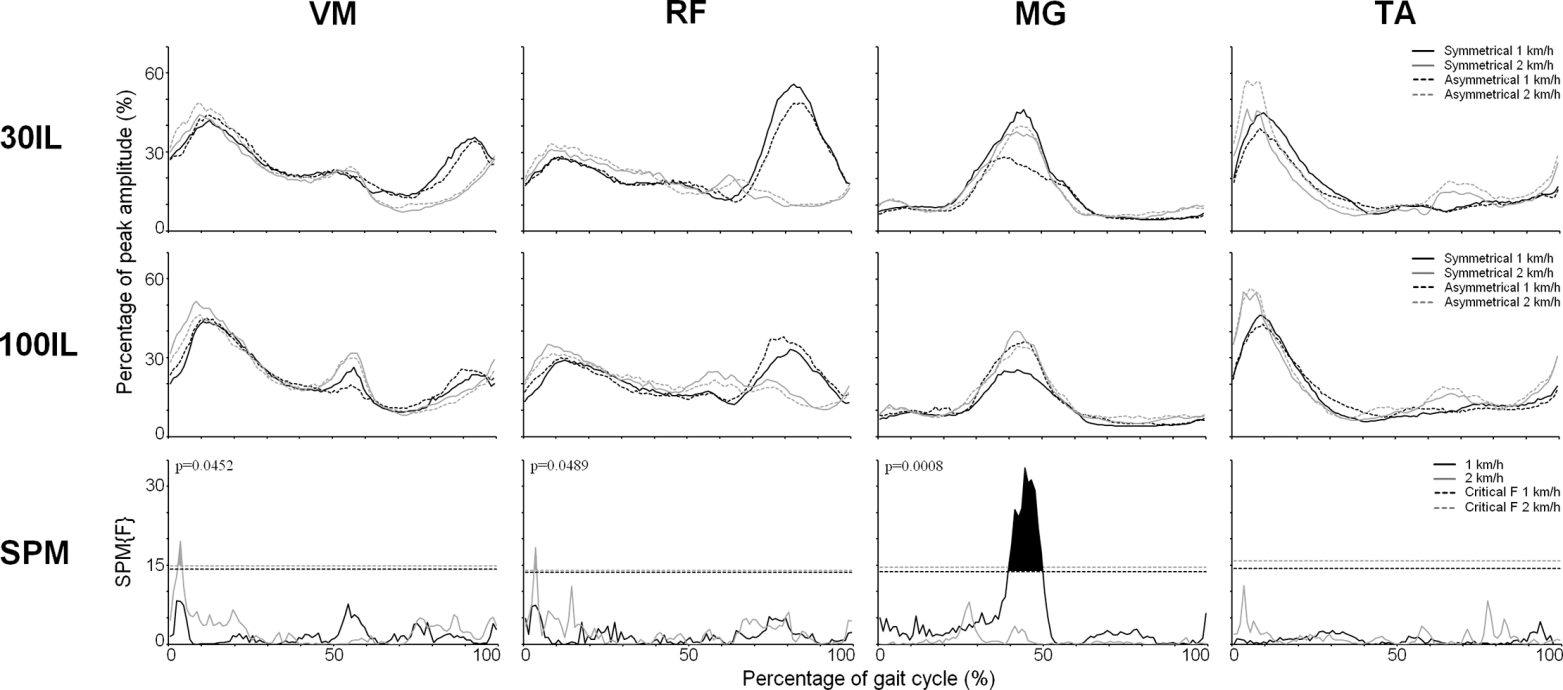
Average EMG profiles for Vastus Medialis (VM), Rectus Femoris (RF), Medial Gastrocnemius (MG) and Tibialis Anterior (TA) combined with the SPM results of the SPM analyses for the Guidance by Symmetry interaction. Averaged EMG profiles are presented for Vastus Medialis (VM), Rectus Femoris (RF), Medial Gastrocnemius (MG) and Tibialis Anterior (TA) combined with the results of the SPM analyses for the Guidance by Symmetry interaction. The EMG profiles are time and amplitude normalized during walking in the Lokomat exoskeleton with 30% guidance provided to the ipsilateral leg (first row) and 100% guidance provided to the ipsilateral leg (second row) at 1 km/h (black lines) and 2 km/h (grey lines) for both symmetrical (solid lines) and asymmetrical (dashed lines) conditions. EMG amplitude is expressed as a percentage of peak amplitude recorded over all conditions. The third row of figures display the SPM{F} values of the Guidance by Symmetry interaction at 1 km/h (black lines) and 2 km/h (grey lines). Threshold F values are indicated by dashed black lines (1 km/h) and dashed grey lines (2 km/h). The displayed p-values represent the chance that a supra-threshold cluster of the given cluster size would be observed in random samplings.

The findings of the Guidance by Symmetry interaction at 2 km/h in VM and RF were at odds with the results at 1 km/h (see Fig 3). Short supra-threshold clusters for the Guidance by Symmetry interaction were found in VM (2.3–3.6%; CT = 14.0, p = 0.0452) and RF (2.6–3.4% CT = 14.0, p = 0.0489). However, the nature of these effects was different from those observed at 1 km/h, as ipsilateral muscle amplitudes decreased when the contralateral leg received less guidance (100IL/30CL vs 100IL/100CL).

## Discussion

The present study addressed the effects of contralateral guidance levels on ipsilateral muscle activity provided by the Lokomat exoskeleton in a group of able-bodied walkers. The aim was to determine if and to what extent ipsilateral muscle activity depends on the level of robotic guidance provided to the contralateral leg. The results show that, when ipsilateral guidance was held constant, ipsilateral muscle activity in MG and GM at 1 km/h was inversely related to the amount of guidance offered to the contralateral leg. These effects were observed in selected muscles at 1 km/h, but not at 2 km/h. The here established short-term effects demonstrate that during walking in the Lokomat exoskeleton, the activity of specific muscles in the ipsilateral leg can be affected by the sensorimotor state of the contralateral leg. The results urge further research on the long-term effects and the use of asymmetrical movement guidance in patient groups targeted for robot assisted gait training.

Robotic gait trainers like the Lokomat offer an alternative for manual assistance through therapists by using actuated exoskeletons to guide stepping, so as to reduce the amount of active muscular contributions. Conform this notion, and in accordance with previous work [12], at 1 km/h the amplitude of muscle activity decreased with a general increase of guidance in 4 of the 8 muscles (as indicated by the main effects of Guidance). At 2 km/h, no systematic general effects of guidance were observed, confirming earlier research on Lokomat guided walking [12] showing that effects of guidance are strongly attenuated at higher treadmill speeds. Crucial to the aims of our study, at 1 km/h, the level of ipsilateral activity in GM and MG depended on the amount of guidance that was provided to the contralateral leg. More specifically, when the ipsilateral leg received 100% guidance, activity in the muscles increased when contralateral guidance was set to 30%, compared to the symmetrical situation (100IL/100CL). Conversely, when the ipsilateral leg was provided 30% guidance, activity was reduced when 100% guidance was provided to the contralateral leg, relative to the symmetrical situation (30IL/30CL). For very short epochs (approximately 1% of the gait cycle), opposite effects were found at 2 km/h in VM and RF: lower and higher levels of contralateral guidance resulted in an inhibition and facilitation, respectively, of activity in the ipsilateral leg. Taken together, these results show that tuning the level of contralateral guidance can influence the amount of ipsilateral muscular effort.

The bilateral couplings involved in human walking become particularly evident when different task constraints are imposed on each of the individual legs, e.g. when more muscle activity is required from one leg than from the other leg (see e.g. [17,18,21–23]). Here, we imposed such an asymmetry by setting the amount of robotic guidance for each of the legs independently. With regard to the functional significance of the observed bilateral effects, it is important to note that the effects at 1 km/h occurred within time regions roughly corresponding to ipsilateral single support, suggesting that the observed inhibition and facilitation of activity was coupled to the muscular efforts to control the contralateral swing leg. During unrestrained (overground or treadmill) walking, swing leg motion is primarily regulated through its passive dynamics, reducing the need for active muscular control [30]. However, as the compensatory torques generated by the Lokomat actuators do not fully eliminate interaction torques due to exoskeleton inertia, friction, and gravity [31], this may necessitate a more active control of the swing limb during Lokomat guided walking, in particular at low guidance settings [9,11]. The data seem to confirm this, as prominent RF activity (which serves to flex the hip and extend the knee) was observed during the ipsilateral swing phase selectively at 1 km/h, and this activity was significantly attenuated when more guidance was provided in general. In addition, as MG is known to control contralateral step length [32], an increase in ipsilateral MG activity during 100IL/30CL compared to 100IL/100CL may be facilitated by the requirement of more active control of the swing limb to overcome inertia. Therefore, it cannot be excluded that the facilitatory effects of lowered contralateral guidance in GM and MG are an indirect result of uncompensated exoskeleton inertia when little movement support is provided.

The functional interpretation of the effects observed at 2 km/h (i.e. a facilitation of early stance activity in VM and RF as a result of lowered contralateral guidance) is not straightforward. Because, consistent with the literature [12], no main effects were detected for Guidance at 2 km/h in these muscles, it can be excluded that these effects were directly related to the imposed guidance manipulations. An explanation why no facilitatory effects of lowered contralateral guidance were found at 2 km/h might be related to attenuated exoskeleton inertia at 2 km/h. Since no consistent effect of guidance is present at 2 km/h, it seems likely that swing control at higher speed levels is less affected by the inertia of the exoskeleton and relies more on passive dynamics like in unrestrained walking. In this situation, the imposed asymmetry of guidance does not affect ipsilateral leg control via bilateral couplings. Lastly, it is important to note that, although the effects of contralateral guidance on the ipsilateral leg were consistent between participants, their magnitude was small and they appeared only briefly (see Fig 3.).

Although the here reported effects of asymmetrical guidance were quite subtle, the observed facilitatory effects may stimulate active contributions by patients during robot assisted gait. Active participation of trainees is an important prerequisite for motor learning and effective training to occur [33,34]. Because robotic guidance is known to reduce the need for muscular effort [12,13], the here reported facilitatory effects of asymmetrical guidance may be particularly desirable. Particularly when patients with unilateral gait disturbances require full robotic guidance to support their affected leg, the required neuromuscular activity necessary for effective training may be stimulated by reducing the guidance level for the unaffected side. Arguably, robotic gait trainers with more advanced control strategies that allow control of the resistance of exoskeleton segments (for example exoskeletons based on force field control see e.g. [35]) may offer possibilities to produce more dramatic effects of asymmetrical support settings.

Generalization of the present results to clinical training situations, e.g. for patients with asymmetrical gait patterns, is not self-evident. First, there are indications that in patients with supraspinal lesions, asymmetrical activation of the legs may result in abnormally coordinated and exaggerated activations, due to diminished supraspinal inhibitory control over spinal pattern generating networks [17,18,36]. Second, the facilitating effects of lowered contralateral guidance were only observed at 1 km/h and not at 2 km/h, indicating that specific speed settings may be required during clinical training situations for a training effect to occur. Thirdly, in the present protocol, no foot straps or BWS were provided. However, in clinical practice, such measures are often applied to support foot lift and weight bearing for patients. As both foot straps and BWS may affect neuromuscular task demands (e.g. BWS is known to generally attenuate muscle activity during Lokomat guided walking [11,12]), generalization of the here observed effects to clinical situations is not straightforward, and requires further inquiry. Lastly, inspection of the present results shows that (facilitatory or inhibitory) effects of asymmetrical guidance during Lokomat walking, may occur in regions of the gait cycle where no activity is found during unrestrained walking. Arguably, these bilateral effects may elicit inappropriate and confusing afferent and/or efferent information, and could potentially induce functionally inappropriate training stimuli. However, it must also be noted that the effects of asymmetrical guidance could be prominently and reliably induced during the stance phase in MG. Training the plantarflexors is important in e.g. stroke, as weakness in this muscle group has been shown to be a main limiting factor for functional gait performance [37,38]. Taken together, the present results should encourage further research on the use of asymmetrical guidance in robotic exoskeletons in patient groups.

## Conclusions

To the best of our knowledge, this is the first study to assess the effect of asymmetrical robotic guidance in able-bodied gait. The results demonstrate that muscle output can be selectively facilitated or inhibited by tuning the guidance provided to the contralateral leg at specific speed settings. More research is needed to establish if asymmetrical guidance settings for robotic exoskeletons may be a useful addition to clinical gait training.

## Supporting information

S1 Data Data of the group averaged EMG profiles and the F values calculated with SPM. The supplementary file contains a separate worksheet for eight muscles: Erector Spinae (ES), Gluteus Medius (GM), Biceps Femoris (BF), Semitendinosus (ST), Vastus Medialis (VM), Rectus Femoris (RF), Medial Gastrocnemius (MG) and Tibialis Anterior (TA). The worksheets display the group-averaged EMG profiles for each symmetrical (30IL/30CL and 100IL/100CL) and each asymmetrical (30IL/100CL and 100IL/30CL) guidance condition together with the SPM{F} values calculated for the Guidance by Symmetry interaction.
(XLSX)
